# Supplementary material for: Health Behaviors in Austrian Apprentices and School Students during the COVID-19 Pandemic
Source: Int J Environ Res Public Health. 2022 Jan 18;19(3):1049. doi: 10.3390/ijerph19031049 (PMC8834496; doi:10.3390/ijerph19031049)
Supplement: Supplementary file 1 [file ijerph-19-01049-s001.zip › ijerph-1542562-supplementary.pdf]

Table S1. Percentage of respondents in each vaccine willingness category, according to each of the health behaviors.

	Definitely	Probably	Rather not	Definitely not	I don't know	*p*-value
Smoking						
Never	42.6%	17.2%	12.5%	12.5%	15.1%	$p = 0.08$
Less than 1x per week	43.1%	23.6%	12.5%	13.9%	6.9%	
1x or more per week	52.4%	16.7%	9.5%	11.9%	9.5%	
Daily	26.5%	15.1%	16%	25.2%	17.2%	
Alcohol						
None	36.4%	17.3%	13.1%	16.6%	16.6%	$p = 0.12$
1-2 days	36.7%	19.2%	12.6%	16.8%	14.7%	
3-5 days	47.2%	15.6%	16.5%	10.1%	10.6%	
6-9 days	40%	19.1%	13.6%	9.1%	18.2%	
10-19 days	47.6%	14.3%	6.3%	17.5%	14.3%	
20-29 days	48.3%	6.9%	6.9%	20.7%	17.2%	
30 days	18.8%	12.5%	12.5%	50%	6.3%	
Cannabis						
None	39.7%	17.3%	13.1%	14.9%	14.8%	$p = 0.85$
1-2 days	39.2%	17.6%	13.7%	17.6%	11.8%	
3-5 days	50%	21.4%	7.1%	7.1%	14.3%	
6-9 days	50%	16.7%	16.7%	16.7%	0%	
10-19 days	44.4%	11.1%	0%	5.6%	38.9%	
20-29 days	33.3%	16.7%	11.1%	22.2%	16.7%	
30 days	20%	6.7%	33.3%	40%	0%	
Exercise						
Mean days per week (SD)	2.12 (1.5)	2.23 (1.64)	2.64 (1.93)	2.94 (2.28)	2.82 (2.24)	$p = 0.42$
Health status						
Excellent	42.6%	15.8%	11.9%	17.4%	12.3%	$p = 0.87$
Good	38%	20.5%	12.2%	13%	16.3%	
Quite good	39.9%	14.1%	14.9%	17.3%	13.7%	
Bad	36.9%	11.9%	17.9%	14.3%	19%	
Group						
Apprentice	25.75%	15.8%	15.3%	23.45%	19.7%	$\mathbf{p < 0.0001}$
School student	53.46%	18.5%	11%	7.1%	9.9%	
